# Molecular Cloning and Characterization of a Wild Eggplant *Solanum aculeatissimum* NBS-LRR Gene, Involved in Plant Resistance to *Meloidogyne incognita*

**DOI:** 10.3390/ijms19020583

**Published:** 2018-02-15

**Authors:** Xiaohui Zhou, Jun Liu, Shengyou Bao, Yan Yang, Yong Zhuang

**Affiliations:** Jiangsu Key Laboratory for Horticultural Crop Genetic Improvement, Institute of Vegetable Crops, Jiangsu Academy of Agricultural Sciences, Nanjing 210014, China; xhzhou1984@sina.com (X.Z.); kehl_lau@foxmail.com (J.L.); baoshengyou@126.com (S.B.); yzyangyan890618@163.com (Y.Y.)

**Keywords:** *Solanum aculeatissimum*, root-knot nematode, resistance, virus-induced gene silencing

## Abstract

Root-knot nematodes, *Meloidogyne* spp., cause considerable damage in eggplant production. Transferring of resistance genes from wild relatives would be valuable for the continued improvement of eggplant. *Solanum aculeatissimum*, a wild relative of eggplant possessing resistance to *Meloidogyne incognita*, is potentially useful for genetically enhancing eggplant. In the present study, we have isolated and characterized a nucleotide-binding site leucine-rich repeat (NBS-LRR) resistance gene, designated as *SacMi*. The full-length cDNA of the *SacMi* gene was obtained using the technique of rapid-amplification of cDNA ends (RACE). The open reading frame of the *SacMi* gene was 4014 bp and encoded a protein of 1338 amino acids. Sequence analysis indicated that *SacMi* belong to the non- Toll/Interleukin-1 receptor (TIR)-NBS-LRR type disease-resistance genes. Interestingly, quantitative RT-PCR showed that *SacMi* is expressed at low levels in uninfected roots, but was up-regulated by infection with *M. incognita*. To investigate the role of *SacMi* in *S. aculeatissimum* resistance against *M. incognica*, the tobacco rattle virus (TRV)-mediated virus-induced gene silencing (VIGS) system was used. Silencing of *SacMi* enhanced susceptibility of *S. aculeatissimum* plants to *M. incognita*, suggesting the possible involvement of *SacMi* in resistance against *M. incognita* infection.

## 1. Introduction

Root-knot nematodes (RKNs), *Meloidogyne* spp., are major parasites of vegetables, causing great economic losses throughout the world, which is especially damaging to solanaceous crops [1,2]. Among *Meloidogyne* species, *Meloidogyne incognita*, *Meloidogyne arenaria*, *Meloidogyne javanica*, and *Meloidogyne hapla* are four economically important and destructive species worldwide [1,3]. The parasites infect plant roots and induce the development of ‘giant cells’. These alterations greatly affect nutrient partitioning and water uptake in the host plants, resulting in wilted and stunted plants with significantly reduced yield [4,5,6]. Resistance to root-knot nematode has been identified in several crops or their wild relatives, such as wild tomato, sweet potato and pepper [5,6,7,8,9]. The well-known *Mi-1* gene, conferring resistance to the three most economically important root-knot nematode species, *M. incognita*, *M. arenaria* and *M. javanica*, was isolated using a positional mapping approach from a wild relative of cultivated tomato and has been widely used in modern processing tomato cultivars [5,10,11]. This single dominant gene shares several structural motifs with other R genes, including nucleotide-binding site (NBS) and leucine-rich repeat (LRR) domains, which are characteristics of a family of plant proteins that are required for resistance against viruses, bacteria, fungi and nematodes [5,6].

Cultivated eggplant (*Solanum melongena*) belongs to the *Solanaceae* family, is an important vegetable crop and is of substantial economic importance in Asia, Africa, and the subtropics [12,13]. However, its production is severely threatened by numerous abiotic or biotic stresses, particularly the root-knot nematodes. According to a conservative estimate, RKNs could cause a 16.67% yield decline in eggplant production in India, which translates into almost $23 million annual monetary loss [14]. The broad host range of *Meloidogyne* spp. makes crop rotation less effective and, due to the adverse effects associated with the use of chemical nematicides, developing plant resistant lines is highly desirable for controlling root-knot nematodes [15,16]. Resistance to root-knot nematodes has been identified in some wild relatives of eggplant [17]; however, due to the cross-incompatibility problem between wild *Solanum* species and *S. melongena*, research of resistance to RKNs is rather limited.

*S. aculeatissimum*, a wild relative of cultivated eggplant, is a well-known economically important plant, and is widely cultivated as a medicinal crop, possessing some desirable characteristics, especially the resistance to soil-borne diseases such as verticillium wilt and root-knot nematode [13,18,19,20]. The successful hybridization between *S. aculeatissimum* and *S. melongena* provides the opportunity to utilize resistance genes from *S. aculeatissimum* [21,22]. In a previous study, we obtained a partial NBS-LRR class resistance gene sequence from transcriptome sequencing of *S. aculeatissimum*, which had a high similarity with *Mi-1* gene of tomato [20]. Therefore, in the present study, the full length of this NBS-LRR gene from *S. aculeatissimum* was cloned by RT-PCR and RACE methods, and its expression pattern against *M. incognita* infection was also analyzed. These findings will be valuable for better understanding and utilizing of resistance genes in *S. aculeatissimum*.

## 2. Results

### 2.1. Isolation and Characterization of SacMi

A partial cDNA sequence of a NBS-LRR resistance gene was previously obtained from transcriptome sequencing of *S. aculeatissimum* [20]. To obtain the full-length cDNA sequence, subsequently 5′-RACE and 3′-RACE assays were carried out. The full-length cDNA was obtained and designated as *SacMi*. The open reading frame (ORF) of *SacMi* is 4014 bp in length and encodes for a protein with 1338 amino acid residues. The molecular mass of the predicted protein is 153.197 kD, and the isoelectric point was calculated to be 4.93. Blastp analysis performed against the PR-proteins in the PRGdb database [23] showed that the *SacMi* was most similar to the *Mi-1.2* and *Rpi-blb2* (74.21% identity at amino acid level). 

**Figure 1:**
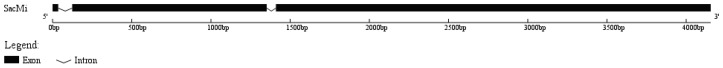
Gene structure of *SacMi*. Exons are indicated by the black boxes from the predicted translation start site (ATG) and stop codon (TAG) and introns are shown as fold lines.

Specific primers *SacMi*-F3ORF/*SacMi*-R6ORF spanning the *SacMi* ORF were designed to obtain the corresponding DNA sequence. A DNA fragment 4159 bp in length with a start codon ATG and a stop codon TAG was obtained. Comparative analysis of the cDNA and DNA sequence of *SacMi* transcript region revealed two introns of 88 bp and 57 bp in length, respectively (Figure 1).

### 2.2. Multiple Sequence Alignment and Phylogenetic Analysis

**Figure 2:**
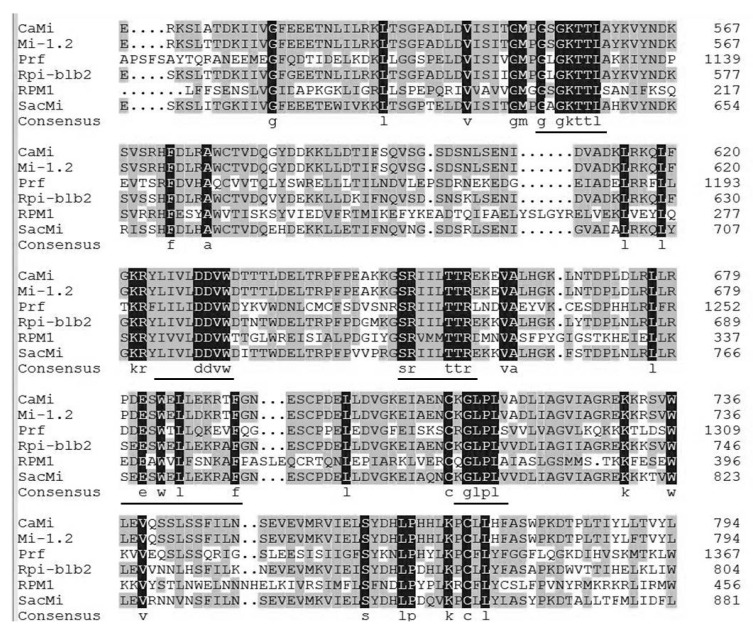
Multiple sequence alignments of the deduced amino acid sequence of *SacMi* and other R proteins, *Mi-1.2* (AAC67238.1), *CaMi* (ABE68835.1), *Prf* (AAC49408.1), *RPM1* (AGC12590.1), *Rpi-blb2* (AAZ95005.1). Nucleotide-binding site (NBS) conserved domains are shaded and indicated by black lines. Black back ground, completely conserved region; Grey back ground, partly conserved region.

Plant NBS-LRR genes can be classified into two types (non-TIR and TIR) based on the presence or absence of a Toll/Interleukin-1 Receptor (TIR) domain at the N-terminus [24]. The NBS domain contains several conserved motifs, including canonical nucleotide-binding kinase 1a or P-loop and Kinase 2 and Kinase 3a, as well as RNBS-A, RNBS-C, GLPL, RNBS-D and MHD. Some are specific to the non-TIR or the TIR-NBS-LRR subfamily, such as RNBS-A-nonTIR and RNBS-D-nonTIR in the nonTIR subclass, and RNBS-A-TIR and RNBS-D-TIR in the TIR subclass [24,25]. Multiple alignments of *SacMi* and other known R genes, including root knot nematode resistance gene *Mi-1.2* and *CaMi* from *Solanum lycopersicum* and *Capsicum annuum*, respectively, as well as *Solanum lycopersicum Prf*, *Arabidopsis thaliana RPM1*, *Solanum bulbocastanum Rpi-blb2,* were performed. The presence of conserved NBS domains such as P-loop, Kinase 2, RNBS-A, RNBS-B, GLPL, and the C-terminus LRR domain was revealed in *SacMi* (Figure 2). Particularly, the presented RNBS-A motifs FDLxKxWVSVSDDF and the amino acid residue Trytophan (W) at the end of Kinase 2 indicated that *SacMi* belongs to the non-TIR subclass of NBS-LRR resistance genes. Structure analysis of the *SacMi* protein sequence revealed that the N-terminal region of the predicted protein contained a potential coiled-coil region.

**Figure 3:**
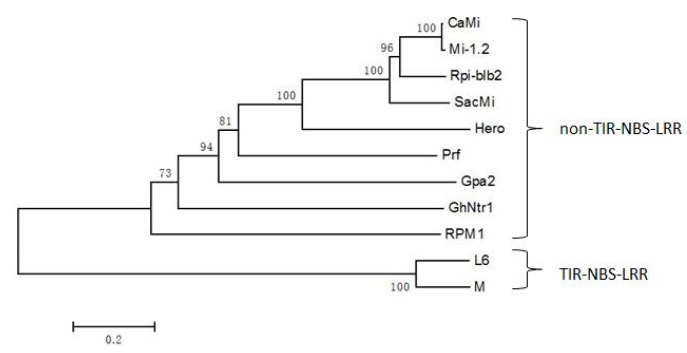
Phylogenetic relationships between *SacMi* and *Mi-1.2* (AAC67238.1), *CaMi* (ABE68835.1), *Prf* (AAC49408.1), *Hero* (CAD29729.1), *RPM1* (AGC12590.1), *Rpi-blb2* (AAZ95005.1), *Gpa2* (AAF04603.1), *GhNTR1* (AIR09519.1), *L6* (AAA91022.1), *M* (AAB47618.1) based on amino acid sequences.

To further understand the evolutionary relationships among *SacMi* and other plant resistance genes, including *Solanum lycopersicum Mi-1.2* (AAC67238.1), *Capsicum annuum CaMi* (ABE68835.1), *Solanum lycopersicum Prf* (AAC49408.1), *Solanum lycopersicum Hero* (CAD29729.1), *Arabidopsis thaliana RPM1* (AGC12590.1), *Solanum bulbocastanum Rpi-blb2* (AAZ95005.1), *Solanum tuberosum Gpa2* (AAF04603.1), *Gossypium hirsutum GhNTR1* (AIR09519.1), *Linum usitatissimum L6* (AAA91022.1), and *Linum usitatissimum M* (AAB47618.1), a phylogenetic tree was constructed based on the amino acid sequences. As revealed in Figure 3, all resistance genes were clustered into two main groups: the non-TIR-NBS-LRR class and the TIR-NBS-LRR class. In the non-TIR-NBS-LRR group, *SacMi* was grouped together with *Mi-1.2*, *CaMi*, and *Rpi-blb2*, and shared a high homology with *Rpi-blb2*.

### 2.3. Expression Analysis of SacMi

**Figure 4:**
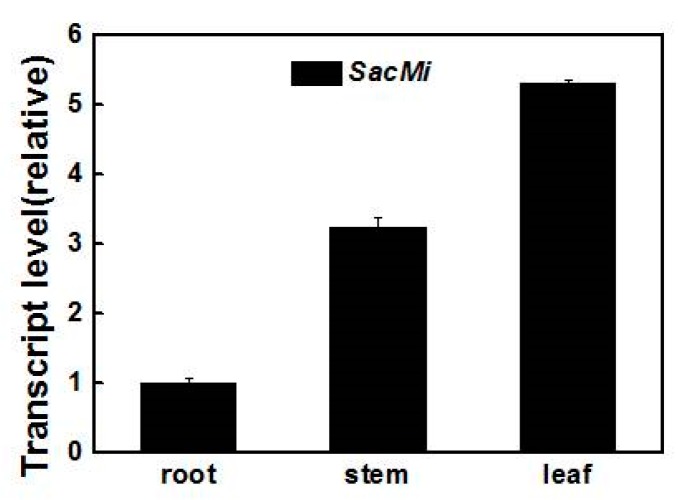
Expression of *SacMi* gene in different tissues of *S. aculeatissimum*.

The expression profile of *SacMi* in different tissues was analyzed by quantitative real-time PCR (qRT-PCR). The results showed that *SacMi* expressed in all tissues of leaf, stem and root (Figure 4). The highest expression level of *SacMi* was detected in leaf tissue, while the expression level of *SacMi* was relatively low in root tissue.

**Figure 5:**
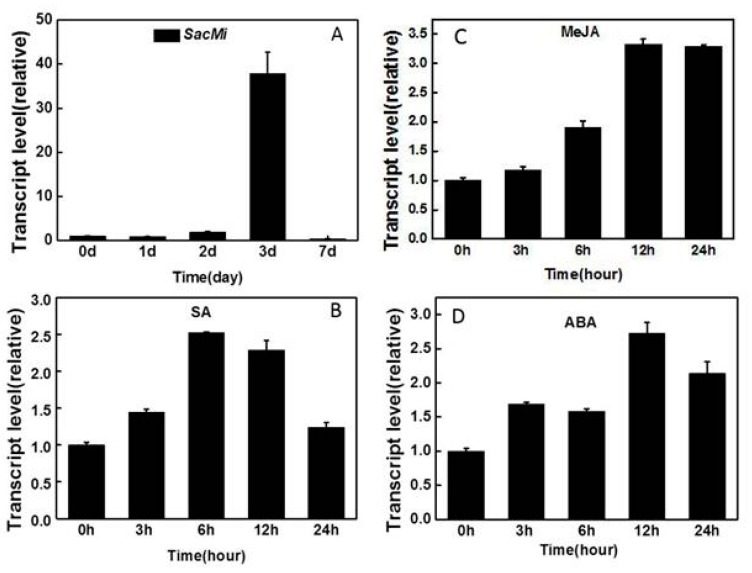
Expression profiles of *SacMi* gene in response to *M. incognita* inoculation (**A**); SA treatment (**B**); MeJA treatment (**C**); and ABA treatment (**D**).

The induction of *SacMi* expression in response to *M. incognita* and defense signaling molecules was also analyzed by qRT-PCR. Following infection by *M. incognita*, the expression of *SacMi* was induced and peaked at 3 d after inoculation with 37.86-fold increase. However, the expression level of *SacMi* was dropped to 0.38-fold at 7 d after inoculation (Figure 5A).

For salicylic acid (SA) treatment, the *SacMi* expression was increased and reached a peak at 6 h with a 2.53-fold increase (Figure 5B). For methyl jasmonate (MeJA) treatment, the *SacMi* expression was gradually induced and reached a maximum at 12 h with a 3.32-fold increase (Figure 5C). For abscisic acid ABA treatment, the *SacMi* expression was also induced and peaked at 12 h with a 2.73-fold increase (Figure 5D).

### 2.4. Silencing of SacMi in S. aculeatissmum Using TRV-VIGS

**Figure 6:**
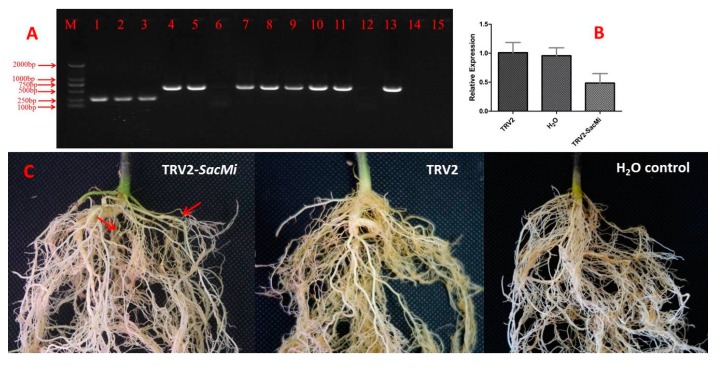
VIGS of *SacMi* increases the sensitivity to *M. incognita* in *S. aculeatissimum*. (**A**) PCR detection of TRV2-*SacMi* and TRV transcripts in vivo. M: Marker, 1–3: plants inoculated with TRV2; 4–13: plants inoculated with TRV2-*SacMi*; 14–15: H_2_O control; (**B**) qRT-PCR analysis of *SacMi* mRNA transcript levels in *SacMi*-silenced plants, empty TRV2 vector control plants and H_2_O control plants; (**C**) Symptoms of TRV2-*SacMi* or TRV2 silenced or H_2_O control *S. aculeatissimum* plalnts infected by *M. incognita* at six weeks. Red arrows indicate developed root galls.

Virus-induced gene silencing (VIGS) provides an alternative approach for functional analysis of genes. The VIGS system has been established in our lab and is proved to be efficient to induce endogenous gene silencing in *S. linnaeanum* [26]. Here, we attempted to silence *SacMi* using tobacco rattle virus (TRV)-mediated gene silencing to determine the role of *SacMi*. To confirm that the TRV had been inoculated into and had multiplied in the *S. aculeatissimum* plants, PCR was performed using RNA extracted from newly developed leaves after 20 d infection with TRV primers (TRV2-F: 5′-TGGGAGATGATACGCTGTT-3′, TRV2-R: 5′-CCTAAAACTTCAGACACG-3′). Viral expression was present in the plants inoculated with TRV2 and TRV2-*SacMi*, and was absent in the plants treated with H_2_O (Figure 6A), which showed that TRV infected the seedlings, transmitted and multiplied from the infiltrating point to other leaves.

qRT-PCR of the transcript levels of *SacMi* gene in inoculated plants were performed to determine the extent of silencing. RNA extracted from infected leaves after 20 d infection of *S. aculeatissimum* agroinfiltrated with empty vector TRV, TRV-*SacMi*, and H_2_O control were used for qRT-PCR. As shown in Figure 6B, the transcript levels of *SacMi* mRNA in the plants infiltrated with TRV-*SacMi* were reduced compared to the control plants (plants infiltrated with empty vector and H_2_O).

The resistance of TRV-*SacMi S. aculeatissimum* plants under *M. incognita* infection was evaluated to determine the possible functional role of *SacMi*. Six weeks after nematode inoculation, root galls developed on the plants silenced with *SacMi*, while no root gall was found on the plants agroinfiltrated with an empty TRV vector or H_2_O, indicating that silencing of *SacMi* leads to increased susceptibility to *M. incognita* infection in *S. aculeatissimum* plants (Figure 6C).

## 3. Discussion

Wild species are rich sources of genetic diversity for disease resistance in domesticated plants [27,28]. The fundamental strategy for controlling RKNs in eggplant is the isolation of resistance genes from their resistant relatives to be used in conventional breeding, genetic engineering and biotechnological approaches. *S. aculeatissimum* possesses resistance to root-knot nematode, would be a valuable source for eggplant improvement. Based on a partial NBS-LRR gene sequence, which was previously obtained from transcriptome sequencing of *S. aculeatissimum*, we were able to successfully clone a root-knot nematode resistance gene *SacMi*, and the structural and phylogenetic analyses of *SacMi* were presented in this study.

Sequence analysis revealed that *SacMi* belongs to the NBS-LRR family of plant resistance genes. The deduced protein sequence of *SacMi* ORF contained typical NBS domains including P-loop, RNBS-A-nonTIR, Kinase-2, RNBS-B, RNBS-C, GLPL, RNBS-D-nonTIR. The conserved NBS region resembles an ATPase domain present in proteins regulating programmed cell death [29,30]. The C-terminus LRR domain is considered to be an effector-binding domain, and has been hypothesized to participate in specific recognition of pathogen effector molecules [31]. Evidence is accumulating that NBS-LRR motifs are common in nematode R-genes [2]. *Mi-1*, *Hero*, *Gpa2*, *Gro1-4*, *Cre3* and *CaMi* have been cloned and all belong to the NBS-LRR gene family [5,6,32,33,34,35]. The exception is for *Hs1^pro-1^*, a nematode resistance gene in sugar beet. This much smaller gene encodes a 282-amino acid protein with an N-terminal LRR and a putative membrane-spanning segment [36].

Most plant resistance genes cloned have been reported to be expressed constitutively [36], such as *Mi* [5] and *Gpa2* [33]. In this study, the expression patterns of *SacMi* were investigated to gain insight into its involvement in *M. incognita* resistance. The *SacMi* is expressed at low levels in uninfected roots of *S. aculeatissimum*, but infection with *M. incognita* significantly enhanced the expression level of *SacMi*, indicating that *SacMi* might be associated with the *M. incognita* resistance in *S. aculeatissimum*. Similar expression profiles have been observed for *Xa1*, a bacterial resistance gene from rice [37], the *pib* rice blast resistance gene [38,39], and *Hs1^pro-1^*, a nematode resistance gene in sugar beet [36]. 

Phytohormones such as salicylic acid (SA), jasmonate (JA), and abscisic acid (ABA) are important regulators in the complex signaling cascades and are involved in the defense responses [40]. SA and JA signaling pathways have been demonstrated to be mutually antagonistic [41], and the ABA has mostly been considered to act as a negative regulator of disease resistance [42]. SA was reported to be an important component of the signaling that leads to *Mi-1*-mediated defense response to root-knot nematode in tomato [43], while JA-dependent signaling does not play a role in *Mi-1*-mediated defense, but an intact JA signaling pathway is required for tomato susceptibility to RKNs [44]. In *Arabidopsis*, SA treatment increased the expression of *SSI4*, which encoded a putative protein belonging to the TIR-NBS-LRR class of R proteins [45]. However, there are also some opposite cases, like *Hs1^pro-1^*, a nematode resistance gene in sugar beet, was not induced by external stimuli including SA, JA, ABA and gibberellic acid (GA). In this study, *SacMi* was activated not only by SA but also by MeJA and ABA, suggesting *SacMi* may play a potential role in mediating cross-talk between defense-signaling pathways [36]. The exact role of these hormones and the cross-talk between them during the defense response to RKNs still remains to be discovered.

A number of resistance genes to various plant pathogenic nematodes have been identified in several crops or their wild relatives [5,6,32,33,34,35,46]. However, the resistance spectrum and genetic properties of these genes are quite different. The previous *Mi-1* gene discovered in tomato is temperature sensitive, resistance mediated by the *Mi-1* gene is compromised at soil temperatures above 28 °C [47]. Hare (1956) identified a dominant root knot nematode resistance gene *N* in *C. frutescens*, but its ability to confer resistance is rendered ineffective at temperatures over 28 °C [48]. Temperature sensitivity appears to be a characteristic of several root-knot nematode R genes in other crop species, including alfalfa [49], sweet patato [50], and cotton [51]. Whether the *SacMi* gene is also sensitive to high temperature requires further study. In addition, the *Mi-1* gene also confers resistance against some isolates of the potato aphid (*Macrosiphum euphorbiae*) and the sweet potato whitefly (*Bemisia tabaci*) [11,52]. Although the partial sequence of *SacMi* gene was obtained from the transcriptome sequencing of *S. aculeatissimum* in response to *Verticillium dahlia*, it was not differentially expressed in the *Verticillium* wilt-treated and control samples. Whether the *SacMi* gene also provides resistance to other plant pathogens needs further investigation. 

This study investigated the existence of root-knot resistance genes in *S. aculeatissimum* and its possible involvement in *M. incognita* disease responses. As root-knot nematodes are becoming more and more severe in China because of the increasing use of greenhouses for eggplant production, development of disease-resistant cultivars is an effective and important way of controlling disease. The successful interspecific hybridization between *S. aculeatissimum* and cultivated eggplant provides the opportunity to transfer useful resistance genes from *S. aculeatissimum* [22]. The development of specific markers of *SacMi* for identification of root-knot nematode resistance in interspecific hybrid progeny will facilitate the introgression of resistant genes from *S. aculeatissimum* to cultivated eggplant.

## 4. Materials and Methods

### 4.1. Full-Length cDNA Cloning

Total RNA was isolated from uninfected roots of *S. aculeatissimum* seedlings using the RNeasy Plant Mini Kit (Qiagen, Hilden, Germany). A partial cDNA sequence obtained from previous transcriptome sequencing of *S. aculeatissimum* [20] was used as template. Extension of the 5′ and 3′ RACE were performed with the specific primers (Table 1). 5′ RACE cDNA and 3′ RACE cDNA were synthesized using 5′/3′ RACE kit, 2nd Generation of Roche and 3′ full race core kit of Takara according to the manufacturer’s instructions, respectively. Based on the full-length cDNA sequence, the genomic DNA was amplified using specific primers *SacMi*-F3ORF/*SacMi*-R6ORF (Table 1).

### 4.2. Bioinformatics Analysis

Homology searches were performed using BLASTX from NCBI [53]. The conserved domain was predicted by the Conserved Domain Database (CDD) [54] from NCBI. The molecular mass and theoretical isoelectric point (pI) were predicted on the ExPASy website [55]. Potential coiled-coil structures were predicted by the COILS program [56,57]. Multiple sequence alignment and phylogenetic tree were carried out by DNAMAN software (version 6.0, Lynnon Biosoft, CA, USA) and MEGA version 6.0 [58,59]. The aligned full-length sequence data were used for the phylogenetic tree using the neighbor-joining method with 1000 bootstraps. Structure of *SacMi* gene was generated using GSDS 2.0 (Center for Bioinformatics (CBI), Peking University, Beijing, China) [60].

### 4.3. Plant Material, Pathogen Inoculation, and Hormone Treatments

*S. aculeatissimum* seeds were sown in sterilized soil and cultured at 25 °C/15 °C, with a photoperiod of 16 h light and 8 h dark in the greenhouse of Jiangsu Academy of Agricultural Sciences, Nanjing, China. The samples of roots, stems and leaves of *S. aculeatissimum* were harvested at the fourth true-leaf stage, prior to exposure to treatments.

For *M. incognita* inoculation, the original culture of *M. incognita* was kindly provided by Professor Chen Jinfeng in the Department of Horticulture at Nanjing Agricultural University. Egg inocula were extracted from infected roots of susceptible tomato cultivar Moneymaker using 0.5% sodium hypochlorite (NaClO) [61]. Seedlings at the fourth true-leaf stage were inoculated with 3000 eggs of *M. incognita* in 3 mL tap water [62]. Control plants were not inoculated but were treated and sampled with distilled water in the same way. The root samples were harvested at 0, 1, 2, 3 d and 7 d post-inoculation.

For hormone treatments, seedlings at the fourth true-leaf stage were sprayed with 2 mM salicylic acid (SA), 100 µM methyl jasmonate (MeJA) and 100 µM abscisic acid (ABA), respectively. Control plants were treated with distilled water in the same way. The leaf samples were collected at the following points: 0, 3, 6, 12 and 24 h.

Three independent biological replications were performed for each treatment mentioned above, and twenty plants were used in each of the replicates. All the freshly collected samples mentioned above were immediately frozen in liquid nitrogen and stored at −80 °C until use. Three independent biological replications were performed for each experiment.

### 4.4. Quantitative Real-Time PCR Analysis

The expression patterns in different tissues (root, stem and leaf) and under *M. incognita* infection, as well as phytohormone treatments, were determined using qRT-PCR. Primers (forward: 5′-CACCCGTGCTTTCCTATTCG-3′ and reverse: 5′-GGGCAGTCTCGCTATTGTTG-3′) were designed using Primer 3 software (version 4.0) [63,64]. The glyceraldehyde-3-phosphate dehydrogenase (GAPDH) gene of eggplant was used as internal control using the following primers, forward: 5′-CCGCTCCTAGCAAAGATGCC-3′ and reverse: 5′-ACCCTCCACAATGCCAAACC-3′. qRT-PCR was performed on the Roche lightcycle 480 system II using a SYBR Green-based PCR assay. Three independent biological replicates of each sample and three technical replicates of each biological replicate were used for qRT-PCR analysis. The reaction containing 10 µL of diluted cDNAs, and 0.4 µL of each primer to a final volume of 20 µL was performed. The PCR conditions were 95 °C for 30 s, followed by 40 cycles of 95 °C for 5 s, 60 °C for 30 s. Relative gene expression levels were calculated using the 2^−ΔΔCt^ [65].

### 4.5. VIGS of SacMi in S. aculeatissimum

The tobacco rattle virus (TRV)-based VIGS system was employed for *SacMi* silencing. The TRV vectors used in this study were described previously [26]. A 330-bp cDNA fragment, spanning the carboxy terminal end of the *SacMi* gene, was cloned into pTRV2 using gene specific primers (forword primer: 5′-CAGGATCCGGCGGATAAGTTCAAGTGC-3′, reverse primer: 5′-GACGGTACCGAGAAAGAAGCAGGCAGAG-3′). The TRV1, empty TRV2, and TRV2-*SacMi* plasmid were then transformed into Agrobacterium tumefaciens strain GV3101, respectively. An equal volume of TRV1 Agrobacterium culture was mixed with empty TRV2 or TRV2-*SacMi* cultures before infiltration. The mixture was infiltrated into cotyledons of 15 d *S. aculeatissimum* plants using 1 mL needleless syringe. Seedlings were maintained for 3 weeks in a growth chamber at 22 °C. At 20 d post-virus inoculation, the plants were infected by *M. incognita*, and then transferred into growth room with a relative humidity of 70%, under a 16/8 h photoperiod.

## Figures and Tables

**Table 1:** Primer sequences used in the study to isolate the *SacMi* gene.

PCR Types	Primers	Primer Sequences (5′-3′)
5′ RACE RT	*SacMi*-R2	GATTTCTCTTCTAAGTCGCTAA
5′ RACE 1st round RT-PCR	*SacMi*-R3	TGTTTCGAGCCCCTGGAGTGCT
5′ RACE 2nd round RT-PCR	*SacMi*-R4	TCAGCATGATACTTGGATAGAT
5′ RACE 2nd round RT-PCR	*SacMi*-R5	TGACGCAACCATTCACCATCAACCTA
3′ RACE 1st round RT-PCR	*SacMi*-F2	TTTCGATCATTGGTATGCCGGGTG
DNA sequence amplification	*SacMi*-F3ORF	ATGGAAAGAGACAAAAGGGAAGC
DNA sequence amplification	*SacMi*-R6ORF	CTAATTAAATAATGGGATATTCATC

## Abbreviations

NBS	Nucleotide binding site
LRR	Leucine-rich repeat
TIR	Toll/Interleukin-1 receptor
TRV	Tobacco rattle virus
VIGS	Virus-induced gene silencing
ORF	Open reading frame
qRT-PCR	Quantitative real-time PCR
SA	Salicylic acid
MeJA	Methyl jasmonate
ABA GA	Abscisic acid Gibberellic acid
RACE	Rapid-amplification of cDNA ends
